# Polymorphism in the Alpha Cardiac Muscle Actin 1 Gene Is Associated to Susceptibility to Chronic Inflammatory Cardiomyopathy

**DOI:** 10.1371/journal.pone.0083446

**Published:** 2013-12-19

**Authors:** Amanda Farage Frade, Priscila Camilo Teixeira, Barbara Maria Ianni, Cristina Wide Pissetti, Bruno Saba, Lin Hui Tzu Wang, Andréia Kuramoto, Luciana Gabriel Nogueira, Paula Buck, Fabrício Dias, Helene Giniaux, Agnes Llored, Sthefanny Alves, Andre Schmidt, Eduardo Donadi, José Antonio Marin-Neto, Mario Hirata, Marcelo Sampaio, Abílio Fragata, Edimar Alcides Bocchi, Antonio Noedir Stolf, Alfredo Inacio Fiorelli, Ronaldo Honorato Barros Santos, Virmondes Rodrigues, Alexandre Costa Pereira, Jorge Kalil, Edecio Cunha-Neto, Christophe Chevillard

**Affiliations:** 1 Heart Institute (InCor), University of São Paulo School of Medicine, São Paulo, São Paulo, Brazil; 2 Institute for Investigation in Immunology (iii), Instituto Nacional de ciencias e tecnologia, São Paulo, São Paulo, Brazil; 3 Laboratory of Immunology, Universidade Federal do Triângulo Mineiro, Uberaba, Minas Gerais, Brazil; 4 Instituto de Cardiologia Dante Pazzanese, São Paulo, São Paulo, Brazil; 5 School of Medicine of Ribeirão Preto, University of São Paulo, Ribeirão Preto, São Paulo, Brazil; 6 Aix-Marseille Université, Marseille, France; 7 Division of Clinical Immunology and Allergy, University of São Paulo School of Medicine, São Paulo, São Paulo, Brazil; Universite de Montreal, Canada

## Abstract

**Aims:**

Chagas disease, caused by the protozoan *Trypanosoma cruzi* is endemic in Latin America, and may lead to a life-threatening inflammatory dilated, chronic Chagas cardiomyopathy (CCC). One third of *T. cruzi*-infected individuals progress to CCC while the others remain asymptomatic (ASY). A possible genetic component to disease progression was suggested by familial aggregation of cases and the association of markers of innate and adaptive immunity genes with CCC development. Since mutations in multiple sarcomeric genes, including alpha-cardiac actin (ACTC1) have been involved in hereditary dilated cardiomyopathy, we investigated the involvement of the ACTC1 gene in CCC pathogenesis.

**Methods and Results:**

We conducted a proteomic and genetic study on a Brazilian study population. The genetic study was done on a main cohort including 118 seropositive asymptomatic subjects and 315 cases and the replication was done on 36 asymptomatic and 102 CCC cases. ACTC1 protein and mRNA levels were lower in myocardial tissue from patients with end-stage CCC than those found in hearts from organ donors. Genotyping a case-control cohort of CCC and ASY subjects for all informative single nucleotide polymorphism (SNP) in the *ACTC1* gene identified rs640249 SNP, located at the 5’ region, as associated to CCC. Associations are borderline after correction for multiple testing. Correlation and haplotype analysis led to the identification of a susceptibility haplotype. Functional assays have shown that the rs640249A/C polymorphism affects the binding of transcriptional factors in the promoter regions of the *ACTC1* gene. Confirmation of the detected association on a larger independent replication cohort will be useful.

**Conclusions:**

Genetic variations at the *ACTC1* gene may contribute to progression to chronic Chagas Cardiomyopathy among *T. cruzi*-infected patients, possibly by modulating transcription factor binding to *ACTC1* promoter regions.

## Introduction

Chagas disease is an infection caused by the protozoan *Trypanosoma cruzi*[1], which is transmitted by an insect vector of the Reduviidae family, blood transfusion or congenitally [1]. The parasite is a major cause of heart disease and cardiovascular death in endemic areas, with approximately 50,000 deaths recorded per year [2]. Chagas disease is particularly prevalent in poor, rural areas of 18 countries in North and South America, ranging from the southern United States to southern Argentina. Despite the implementation of vector control programs in several countries, nine million people are currently infected and 40 million people are still at risk of contracting the infection [3]. Acute *T. cruzi* infection causes acute myocarditis accompanied by blood and tissue parasitism, which is asymptomatic in most cases [4]. A strong innate and adaptive immune response against *T. cruzi* leads to the control of tissue and blood parasitism, but not its complete elimination, resulting in the establishment of low-grade chronic infection [5]. Thirty percent of infected individuals develop chronic Chagas cardiomyopathy (CCC), an inflammatory dilated cardiomyopathy that is, by far, the most important clinical consequence of *T. cruzi* infection. This condition has a fatal outcome and the only treatment is heart transplantation. Ten percent of the patients develop digestive system disease [6]. Most of the other infected individuals remain asymptomatic (ASY) and free from heart disorders for life. Heart failure due to CCC has a worse prognosis and a 50% lower survival rate than cardiomyopathies of –non inflammatory origin, such as ischemic and idiopathic dilated cardiomyopathy [7,8]. CCC is characterized by inflammation and a myocardial remodeling process: T cell/macrophage-rich myocarditis, hypertrophy and fibrosis with cardiomyocyte damage. The myocardial inflammatory infiltrate is thought to play a major role in disease development and progression [9,10]. The myocardial cytokine production profile suggests an IFNγ/TNFα Th1 type response, with interferon γ-induced chemokines [11-16]. Along with the induction of direct inflammatory damage, we have shown that IFNγ directly induces profound changes in cardiomyocyte gene expression, including the hypertrophic program [17]. IL-18 and CCR7 ligands [18-20], CCL2, IL1β and TNF-α [21,22] have also shown to be involved in direct induction of cardiac hypertrophy and/or fibrosis. Evidence from the Syrian hamster model of chronic Chagas cardiomyopathy indicates that while the intensity of inflammation correlated with ventricular dilation (i.e. disease progression), it was not associated to death among hamsters with end-stage dilated chronic Chagas cardiomyopathy [23]. This may suggest that additional, non-inflammatory factors could contribute to severity or progression to death from CCC. Taken together, this suggests that the dilated cardiomyopathy phenotype and clinical outcome may involve an interplay between the inflammatory environment and specific gene regulation in cardiomyocytes and other myocardial cell types.

The mechanisms underlying the differences in progression to chronic Chagas cardiomyopathy are not fully understood. Familial aggregation of cases of chronic Chagas cardiomyopathy [24] suggests a possible genetic component to disease susceptibility. Several polymorphic markers of innate and adaptive immunity genes have been found to be associated with CCC susceptibility (Reviewed in 25-27).

On the other hand, variations in more than 40 genes, most of which encode sarcomeric contractile proteins, or proteins from the cytoskeleton or nuclear lamina, have been shown or proposed to cause dilated cardiomyopathy [28,29]. Effective muscle contraction requires force generation by the sarcomere and force transmission to the cytoskeleton and the extracellular matrix. ACTC1 (alpha-cardiac actin) is the main component of the thin filament of the sarcomere. One end of the polarized actin filament forms crossbridges with myosin, and the other end is immobilized, attached to a Z band or an intercalated disc [30,31]. Thus, actin transmits force between adjacent sarcomeres and neighboring myocytes to effect coordinated contraction of the heart. Olson et al. (1998) found two rare ACTC1 gene mutations that cosegregated with a form of hereditary idiopathic dilated cardiomyopathy [32]. Jiang et al. (2010) found that ACTC1 mRNA and protein levels were significantly lower than those in controls and that this was strongly correlated with cardiomyocyte apoptosis [33]. They also showed that the forced reduction of ACTC1 gene expression in cardiomyocytes led to increased apoptosis. Cardiomyocyte apoptosis is significantly higher in heart failure patients than controls. Increased cardiomyocyte apoptosis has also been reported in Chagas disease [34]. Induction of similar levels of cardiomyocyte apoptosis led to heart failure in murine models; apoptosis is possibly involved in cardiomyocyte loss that may lead to heart failure [35]. Moreover, in syrian hamster model, during the acute phase, the alpha cardiac muscle actin 1 protein was lower in groups from animals without signs of acute-phase infection (-4%) and from animals with signs of acute-phase infection (-19%) compared with pools from Control animals [36]. We investigated the involvement of the *ACTC1* gene in CCC pathogenesis, by comparing *ACTC1* mRNA and protein levels in myocardial tissues from patients with CCC and normal donor hearts, and determining whether *ACTC1* gene polymorphisms were associated with CCC. 

## Materials and Methods

### Ethical standard

Written informed consent was obtained from all the patients, in accordance with the guidelines of the various internal review boards. The protocol was also approved by the INSERM Internal Review Board and the Brazilian National Ethics in Research Commission (CONEP). Written informed consent was obtained from the patients. All the patients enrolled in this study were over 21 years old so it didn’t require parents/guardians provided consent. In the case of samples from heart donors, written informed consent was obtained from their families. Investigations were conformed to the principles outlined in the Declaration of Helsinki.

### Diagnostic Criteria

The diagnostic criteria for Chagas disease included the detection of antibodies against *T. cruzi* in at least two of three independent serological tests (EIA [Hemobio Chagas; Embrabio São Paulo], indirect immunofluorescence assays [IFA-immunocruzi; Biolab Merieux], and indirect hemagglutination tests [Biolab Merieux]) [17]. All Chagas disease patients underwent standard electrocardiography and echocardiography for clinical group stratification. Echocardiography was performed at the hospital, with a Sequoia model 512 echocardiograph with a broad-band transducer. Left ventricular dimensions and regional and global function, including the recording of left ventricular ejection fraction (LVEF), were evaluated with a two-dimensional and M-mode approach, in accordance with the recommendations of the American Society of Echocardiography. Asymptomatic subjects have no electrocardiography and echocardiography changes. CCC patients presented typical conduction abnormalities (right bundle branch block and/or left anterior division hemiblock) [37]. CCC patients with significant left ventricular systolic dysfunction (LVEF <40%) were classified as having severe CCC, whereas those with no significant ventricular dysfunction (LVEF ≥40%) were classified as having moderate CCC. We selected 0.4 as arbitrary cutoff value that has been used to define ventricular dysfunction by several authors and our group [16,38,39]. 

### Myocardial samples

Myocardial samples were obtained from the left ventricular free wall of the hearts of patients with severe CCC and end-stage heart failure, at the time of heart transplantation. Similar samples were obtained from healthy hearts from organ donors not used for transplantation for technical reasons. 

### Analysis of protein expression by Immunoblotting

On myocardial samples from CCC patients (*N*=5) and organ donor controls (NC; *N*=5), proteins extraction and inmmunoblotting were performed according to Teixeira et al. [40]. Briefly, protein homogenates were obtained using lysing solution (1:10 w/v) containing 7 mol/L urea, 10 mmol/L Tris, 5 mmol/L magnesium acetate and 4 % CHAPS, pH 8.0, by mechanical homogenization (PowerGen, Thermo Fisher Scientific, Waltham, USA). Extracts of myocardial samples containing 30 μg of protein were heated for 5 min at 95 °C, and subjected to sodium dodecyl sulfate polyacrylamide gel electrophoresis (SDS-PAGE) using 12.5% polyacrylamide gel and the vertical electrophoresis system Ruby SE600 (GE Healthcare, Waukesha, USA). After electrophoresis, proteins were transferred from gel to a nitrocellulose membrane using the TE Semi-Dry Transfer Unit (GE Healthcare, Waukesha, USA). The nitrocellulose membranes were incubated with monoclonal antibodies: monoclonal anti-actin, alpha cardiac muscle 1 (ACTC1) (Santa Cruz Biotechnology, Inc., Santa Cruz, USA) and polyclonal anti-glyceraldehyde 3-phosphate dehydrogenase (GAPDH) (R&D Systems, Minneapolis, USA). Each membrane was subjected to incubation with compatible secondary antibodies conjugated with peroxidase, developed using ECL Plus Western Blotting Detection Reagents (GE Healthcare) and detection using X-ray equipment. Analysis of densitometry was performed using the program ImageQuant TL (GE Healthcare, Waukesha, USA). Each biological sample was analyzed in a duplicate experiment.

### Analysis of mRNA expression by real-time reverse transcriptase (RT)-PCR

Total RNA from left ventricle samples (5x5x5 mm) was isolated using the RNeasy Fibrous tissue kit (Qiagen, Hilden, Germany). Contaminating DNA was removed by treatment with RNase-free DNase I. cDNA was obtained from 5μg total RNA using Super-script II™ reverse transcriptase (Invitrogen, Carlsbad, USA). mRNA expression was analyzed by real-time quantitative reverse transcriptase (RT)-PCR with SYBR Green I PCR Master Mix (Applied Biosystems, Foster City, USA) and 250nM of sense and anti-sense primers using the ABI Prism 7500 Real Time PCR System (Applied Biosystems, Foster City, USA). The following primers were designed using Primer Express software version 3.0: GAPDH (M33197): (F) 5’-GGTCTCCTCTGACTTCAACA-3’, (R) 5’-AGCCAAATTCGTTGTCATACC-3’; ACTC1 (NM_005159): (F) 5´-CTTCATTGGTATGGAATCTGCTGG-3´, (R) 5´-CATTGTTGGCATACAGGTCCTTG-3´. After every PCR, an amplicon melting point curve was obtained. This yielded a single peak with the expected temperature provided by Primer Express software, confirming the specificity of the PCR. GAPDH mRNA expression was used for normalization [17]. The amount of mRNA in the left ventricles samples was calculated using the 2^-DCt^ method [41] Each biological sample was analyzed in triplicate within the same experiment, Ct average was used in the 2^-DCt^ calculation.

### Study population for polymorphism analysis

The patients and controls were enrolled in Sao Paulo, Minas Gerais and Bahia states. Patients with digestive forms or mixed forms were excluded of this study. Patients were classified as asymptomatic (*n* = 118) or as having CCC (*n* = 315). Asymptomatic individuals were used as the control subjects for this study because they were from the same areas of endemicity as the patients with CCC, had encountered the parasite and had tested seropositive for *T. cruzi* infection, but the infection had not progressed to CCC. Of the 118 asymptomatic subjects, 45.3% were male, whereas in the CCC patients group, this percentage reaches 61.3%. The difference in sex distribution between the groups was significant (*p*=1.21E-4; OR=2.126; 95%CI: 1.450 - 3.12). It is well known that male patients infected with *T. cruzi* have a higher risk of progression to CCC than female patients [42-44]. Of 315 patients with CCC, 106 (42 men [39.6%] and 64 women [60.4%]) showed no significant ventricular dysfunction and were thus classified as having moderate CCC, whereas 199 (144 men [72.4%] and 55 women [27.6%]) had severe ventricular dysfunction and were classified as having severe CCC. Data for left ventricular ejection fraction were missing for 10 patients with CCC. Of 315 patients with CCC, 106 showed no significant ventricular dysfunction and 199 had severe ventricular dysfunction. 

The second cohort focused exclusively on male patients with Chagas disease. This replication cohort included asymptomatic (*n* = 36) and CCC patients (*n* = 102). Of the 102 patients with CCC, 48 had severe ventricular dysfunction (left ventricular ejection fraction <40%).

### Blood samples and DNA preparation

For each subject, 5 to 15 ml of blood were collected into EDTA tube. Genomic DNA was isolated on a silica-membrane according to the manufacturer’s protocol (QIAamp DNA Blood Max Kit, Qiagen, Hilden, Germany).

### SNP selection

Tag single nucleotide polymorphisms (SNPs) were selected on the basis of HapMap Data for the Caucasian and Yoruba reference populations. Tag SNPs were selected within a region extending 5 kb on either side of the candidate gene. The minor allele frequency cut off value was arbitrarily set at 20%. Tag SNPs characterised by a MAF over 20% on at least one reference population were selected. So, we genotyped a total of 18 SNPs. Nine of the markers were located in the promoter region (rs639735, rs640249, rs893130, rs475786, rs893131, rs893132, rs533225, rs670957, rs525720), three were located in intronic regions (rs7166484 (intron2); rs2070664 (intron3); rs3729755 (intron 5)), one was located in the 3’UTR region (rs533021) and five were located in the 3’region of the *ACTC1* gene (rs1851317, rs492038, rs4924215, rs4924214, rs7179902).

### SNP genotyping

Most of the genotyping was done with the Golden Gate genotyping assay (Illumina, San Diego, USA). In some cases, genotyping assays were performed with the TaqMan system (Applied Biosystems, Foster City, USA) according to the manufacturer’s instructions. 

### Statistical analysis

SPSS Statistics software v. 17.0 (IBM, Armonk, USA) was used for statistical analyses. We performed some stepwise binary logistic regression analysis on the whole population, to analyse the relationship between the probability of an individual to develop chronic Chagas cardiomyopathy and the main covariates (sex and polymorphisms). Sex is considered as a binary covariate. For each polymorphism, two alleles were available leading to three different genotypes. In our stepwise binary logistic regression analysis, genotypes were considered as binary covariates. We compare homozygous genotype (usually the more frequent) to the heterozygote genotype + the rare homozygote genotype. We corrected p values according to Nyholt’s procedure for multiple testing correction [45,46]. 

We performed a haplotype reconstruction of the study population based on the SNPs rs640249 and rs641563. This reconstruction was done with HapAnalyzer software (http://ngri.re.kr/HapAnalyzer). We used the PLEM algorithm according to the indications provided with the software. This reconstruction will affect two haplotypes for each individual. As we have two binary markers, four our haplotypes were detected with various frequencies: (**H1**) rs640249**C**-rs641563**C**: 58.8%; (**H2**) rs640249**A**-rs641563**A**: 39.1%; (**H3**) rs640249**C**-rs641563**A**: 1.6%; (**H4**) rs640249**A**-rs641563**C**: 0.5%. This last haplotype is very rare. So, subjects carrying at least on copy of this haplotype were excluded from the analysis by lack of power. We have focused our analyse on the three main haplotypes. Six haplotype combinations were possible (H1H1, H1H2, H1H3, H2H2, H2H3, H3H3). The haplotype combination was considered as a new covariate with six classes.

### Electrophoretic mobility shift assay (EMSA)

Nuclear extracts were prepared from primary cardiomyocyte culture (PromoCell; Heidelberg, Germany). Complementary single-stranded oligonucleotides, containing the study polymorphism surrounding sequence, span 10 bp on either side of the variant nucleotide. For rs640249 polymorphism the sequence was: 5’biotin GTTTGCCCTG **[A/C]** ACGTCTCCCT 3’. For rs641563 polymorphism the sequence was: 5’ biotin TAGACTGCCT **[A/C]** ATAGAAATTC 3’. Nuclear extract and electrophoretic mobility shift assay were done according to a published protocol [47].

## Results

**Figure 1:**
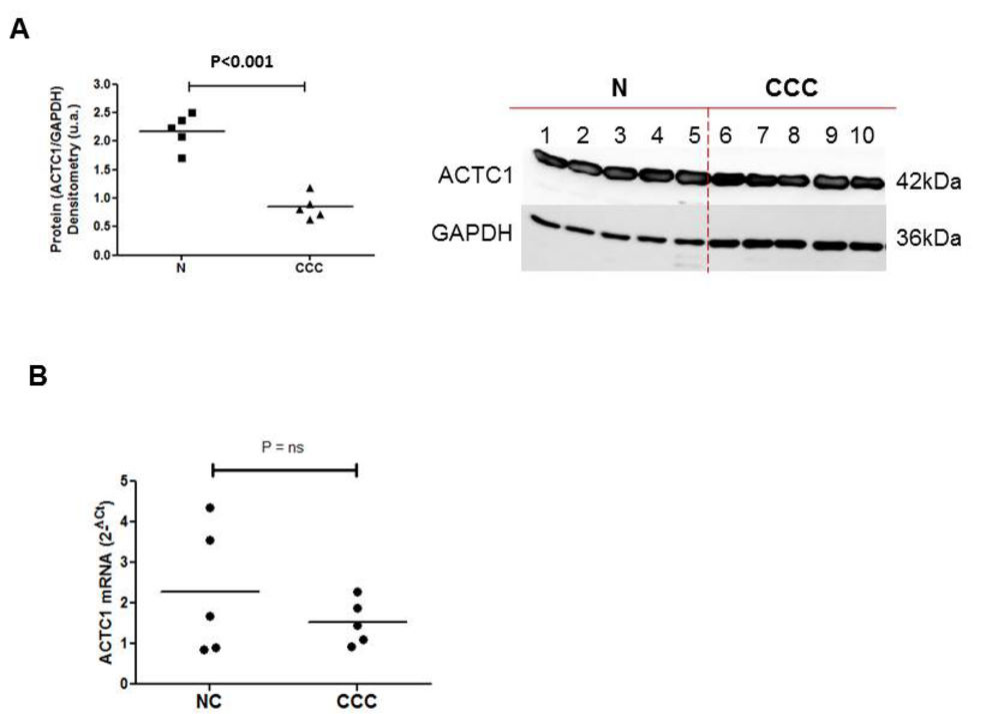
Relative quantification of alpha-cardiac actin 1 (ACTC1) by immunoblotting. Myocardial samples were obtained from the left ventricular free wall of the hearts of patients with severe CCC and end-stage heart failure, at the time of heart transplantation. Samples from five hearts from CCC patients (at least two positive results in three independent anti-*T*. *cruzi* serology tests, as indicated above), and from healthy hearts from organ donors not used for transplantation for technical reasons were used. Immunoblotting and protein quantification were done in duplicate. A. The immunoblot and the protein quantification result of the first experiment are presented here. The central line represents the median. Representative results from two experiments are shown here. A Mann-Whitney test was performed and differences were considered significant if *P*<0.001. **B**. Real-time quantitative PCR was carried out on the same samples. All the samples were tested in triplicate with GAPDH, the expression of which has been shown to vary little between human myocardial tissue samples. Data were normalized and the relative levels of each mRNA were calculated by the 2^-ΔCt^ method.

Alpha-cardiac actin 1 (ACTC1) protein levels in the myocardium from left ventricle free wall differed between CCC patients and normal control (NC hearts) (Table S1), after normalization against GAPDH. Median ACTC1 protein levels were 64 % lower in the myocardium of CCC patients than in myocardial samples from individuals without cardiomyopathies (*p*<0.001) (Figure 1A). The duplicate analysis is provided in Figure S1. On the same samples, we performed a gene expression analysis. Experiments were done in triplicate and we calculated the mean value for each sample. Median ACTC1 mRNA levels were 32% lower in heart samples from CCC patients than in those from controls (Figure 1B), suggesting possible transcriptional control of *ACTC1* levels. However, this difference in ACTC1 mRNA levels was not statistically significant due to high inter individual variation in the normal control group. 

Polymorphism analysis (Table 1) was done on a main cohort including 433 Chagas disease patients born in areas in which the disease is endemic in the states of São Paulo and Minas Gerais. Patients were classified as asymptomatic (*n* = 118) or as having CCC (*n* = 315). Eighteen Tag SNPs were genotyped successfully. The rs1851317 marker was not informative in our study population. The genotype distribution of each informative SNP (17 markers) is summarized in Table 2. All the SNPs were in Hardy-Weinberg equilibrium in asymptomatic subjects (p>0.25). 

**Table 1:** List of the tag SNPs genotyped on the original study population.

**Tag SNP**	**Position relative to coordinate system**	**Position relative to transcription start point**
rs639735 C/T	35091844	-4835
rs640249 C/A	35091686	-4677
rs893130 A/T	35091453	-4444
rs475786 C/G	35091138	-4129
rs893131 T/C	35090912	-3903
rs893132 T/C	35090060	-3051
rs533225 A/T	35089962	-2953
rs670957 G/A	35089432	-2423
rs525720 G/A	35089134	-2125
rs7166484 G/A	35086366	643
rs2070664 A/G	35085201	1808
rs3729755 G/C	35084215	2794
rs533021 G/A	35080931	6078
rs1851317 T/G	35077786	9223
rs492038 T/G	35077112	9897
rs4924215 G/A	35075673	11336
rs4924214 T/C	35075666	11343
rs7179902 A/T	35075531	11478

**Table 2:** Genotype distribution on asymptomatic individuals (ASY) and cases (CCC) taking into account the gender and the left ventricular ejection fraction values.

		**ASY**			**CCC**			**CCC(EF<0.4)**			**CCC(EF>0.4)**		
**SNP**		**Total**	**Male**	**Female**	**Total**	**Male**	**Female**	**Total**	**Male**	**Female**	**Total**	**Male**	**Female**
rs639735C/T	CC	52 (44.8%)	19 (36.5%)	32 (50.8%)	147 (47.9%)	86 (46.0%)	59 (50.0%)	96 (49.0%)	69 (48.6%)	27 (50.0%)	48 (47.1%)	16 (40.0%)	32 (51.6%)
	CT	50 (43.1%)	28 (53.8%)	22 (34.9%)	134 (43.6%)	88 (47.1%)	46 (39.0%)	85 (43.4%)	64 (45.1%)	21 (38.9%)	44 (43.1%)	21 (52.5%)	23 (37.1%)
	TT	14 (12.1%)	5 (9.6%)	9 (14.3%)	26 (8.5%)	13 (7.0%)	13 (11.0%)	15 (7.7%)	9 (6.3%)	6 (11.1%)	10 (9.8%)	3 (7.5%)	7 (11.3%)
rs640249C/A	CC	36 (31.8%)	14 (26.9%)	21 (35.0%)	115 (37.7%)	66 (35.1%)	48 (41.7%)	78 (40.0%)	57 (40.4%)	21 (38.9%)	36 (35.6%)	9 -0,22	27 -0,45
	CA	49 (43.4%)	24 (46.2%)	25 (41.7%)	150 (49.2%)	100 (53.2%)	49 (42.6%)	94 (48.2%)	67 (47.5%)	27 -0,5	49 (48.5%)	27 (65.9%)	22 (36.7%)
	AA	28 (24.8%)	14 (26.9%)	14 (23.3%)	40 (13.1%)	22 (11.7%)	18 (15.7%)	23 (11.8%)	17 (12.1%)	6 (11.1%)	16 (15.8%)	5 (12.2%)	11 (18.3%)
rs893130A/T	AA	91 (77.8%)	38 (73.1%)	52 (81.3%)	230 (73.7%)	144 (75.4%)	85 (71.4%)	145 (73.2%)	108 (75.0%)	37 (68.5%)	76 (73.1%)	37 (68.5%)	30 (73.2%)
	AT	24 (20.5%)	14 (26.9%)	10 (15.6%)	74 (23.7%)	43 (22.5%)	30 (25.2%)	48 (24.2%)	32 (22.2%)	16 (29.6%)	25 -0,24	16 (29.6%)	11 (26.6%)
	TT	2 (1.7%)	0 (0.0%)	2 (3.1%)	8 (2.6%)	4 (2.1%)	4 (3.4%)	5 (2.5%)	4 (2.8%)	1 (1.9%)	3 (2.9%)	1 (1.9%)	0 (0.0%)
rs475786C/G	CC	89 (85.6%)	40 (87.0%)	48 (84.2%)	209 (77.7%)	132 (81.0%)	76 (72.4%)	133 (77.8%)	98 (81.0%)	35 (70.0%)	69 (76.7%)	30 (81.1%)	39 (73.6%)
	CG	15 (14.4%)	6 (13.0%)	9 (15.8%)	59 (21.9%)	30 (18.4%)	29 (27.6%)	37 (21.6%)	22 (18.2%)	15 (30.0%)	21 (23.3%)	7 (18.9%)	14 (26.4%)
	GG	0 (0.0%)	0 (0.0%)	0 (0.0%)	1 (0.4%)	1 (0.6%)	0 (0.0%)	1 (0.6%)	1 (0.8%)	0 (0.0%)	0 (0.0%)	0 (0.0%)	0 (0.0%)
rs893131T/C	TT	81 (68.6%)	36 (67.9%)	45 (70.3%)	195 (62.7%)	117 (61.9%)	77 (64.2%)	127 (64.5%)	89 (62.7%)	38 (69.1%)	63 (60.6%)	25 -0,61	38 (60.3%)
	TC	34 (28.8%)	16 (30.2%)	17 (26.6%)	98 (31.5%)	62 (32.8%)	35 (29.2%)	59 (29.9%)	45 (31.7%)	14 (25.5%)	35 (33.7%)	14 (34.1%)	21 (33.3%)
	CC	3 (2.5%)	1 (1.9%)	2 (3.1%)	18 (5.8%)	10 (5.3%)	8 (6.7%)	11 (5.6%)	8 (5.6%)	3 (5.5%)	6 (5.8%)	2 (4.9%)	4 (6.3%)
rs893132T/C	T/T	60 (54.5%)	26 (54.2%)	33 (54.1%)	186 (60.6%)	111 (59.0%)	73 (62.4%)	121 (62.1%)	86 (61.0%)	35 (64.8%)	59 (57.3%)	23 (54.8%)	36 (59.0%)
	T/C	42 (38.2%)	17 (35.4%)	25 (41.0%)	107 (34.9%)	64 (34.0%)	43 (36.8%)	66 (33.8%)	47 (33.3%)	19 (35.2%)	39 (37.9%)	15 (35.7%)	24 (39.3%)
	C/C	8 (7.3%)	5 (10.4%)	3 (4.9%)	14 (4.6%)	13 (6.9%)	1 (0.9%)	8 (4.1%)	8 (5.7%)	0 (0.0%)	5 (4.9%)	4 (9.5%)	1 (1.6%)
rs533225A/T	AA	90 (86.5%)	41 (89.1%)	48 (84.2%)	207 (84.5%)	132 (87.4%)	74 (79.6%)	134 (86.5%)	96 (88.1%)	38 (75.6%)	66 (79.5%)	32 (84.2%)	34 (75.6%)
	AT	13 (12.5%)	5 (10.9%)	8 -0,14	35 (14.3%)	19 (12.6%)	16 (17.2%)	19 (12.3%)	13 (11.9%)	10 (22.2%)	16 (19.3%)	6 (15.8%)	10 (22.2%)
	TT	1 (1.0%)	0 (0.0%)	1 (1.8%)	3 (1.2%)	0 (0.0%)	3 (3.2%)	2 (1.3%)	0 (0.0%)	1 (2.2%)	1 (1.2%)	0 (0.0%)	1 (2.2%)
rs670957G/A	GG	36 (32.1%)	16 (32.7%)	20 (32.3%)	80 (26.1%)	50 (26.6%)	30 (25.9%)	46 (23.4%)	34 (23.8%)	12 (22.2%)	33 (32.7%)	15 (37.5%)	18 (29.5%)
	GA	51 (45.5%)	22 (44.9%)	29 (46.8%)	149 (48.7%)	89 (47.3%)	58 (50.0%)	103 (52.3%)	71 (49.7%)	32 (59.3%)	40 (39.6%)	15 (37.5%)	25 (41.0%)
	AA	25 (22.3%)	11 (22.4%)	13 -0,21	77 (25.2%)	49 (26.1%)	28 (24.1%)	48 (24.4%)	38 (26.6%)	10 (18.5%)	28 (27.9%)	10 (25.0%)	18 (29.5%)
rs525720G/A	GG	50 (68.5%)	25 (80.6%)	25 -0,61	189 -0,75	113 (73.4%)	74 (77.1%)	118 (73.8%)	83 (72.2%)	35 (77.8%)	64 (75.3%)	26 (74.3%)	38 (76.0%)
	GA	20 (27.4%)	5 (16.1%)	14 (34.1%)	60 (23.8%)	38 (24.7%)	22 (22.9%)	41 (25.6%)	31 (27.0%)	10 (22.2%)	19 (22.4%)	7 (20.0%)	12 (24.0%)
	AA	3 (4.1%)	1 (3.2%)	2 (4.9%)	3 (1.2%)	3 (1.9%)	0 (0.0%)	1 (0.6%)	1 (0.9%)	0 (0.0%)	2 (2.4%)	2 (5.7%)	0 (0.0%)
rs7166484G/A	GG	44 (39.6%)	21 (42.0%)	22 (36.7%)	101 (32.4%)	54 (28.3%)	46 (38.7%)	55 (27.6%)	36 -0,25	19 (34.5%)	42 (40.8%)	16 (39.0%)	26 (41.9%)
	GA	50 (45.0%)	20 (40.0%)	30 (50.0%)	158 (50.6%)	105 (55.0%)	52 (43.7%)	108 (54.3%)	80 (55.6%)	28 (50.9%)	44 (42.7%)	21 (51.2%)	23 (37.1%)
	AA	17 (15.3%)	9 (18.0%)	8 (13.3%)	53 (17.0%)	32 (16.8%)	21 (17.6%)	36 (18.1%)	28 (19.4%)	8 (14.5%)	17 (16.5%)	4 (9.8%)	13 (21.0%)
rs2070664A/G	AA	42 (36.8%)	16 (30.8%)	25 (41.0%)	123 (39.9%)	71 (38.0%)	51 (42.9%)	65 (33.2%)	46 (32.6%)	19 (34.5%)	53 (51.5%)	21 (52.5%)	32 (50.8%)
	AG	61 (53.5%)	33 (63.5%)	28 (45.9%)	143 (46.4%)	90 (48.1%)	52 (43.7%)	102 -0,52	73 (51.8%)	29 (52.7%)	38 (36.9%)	15 (37.5%)	23 (36.5%)
	GG	11 (9.6%)	3 (5.8%)	8 (13.1%)	42 (13.6%)	26 (13.9%)	16 (13.4%)	29 (14.8%)	22 (15.6%)	7 (12.7%)	12 (11.7%)	4 (10.0%)	8 (12.7%)
rs3729755G/C	GG	71 (62.3%)	31 (59.6%)	39 (63.9%)	175 (55.9%)	104 (54.5%)	70 (58.3%)	113 (56.8%)	80 (55.6%)	33 (57.1%)	57 (54.8%)	21 (51.2%)	36 (57.1%)
	GC	35 (30.7%)	17 (32.7%)	18 (29.5%)	121 (38.7%)	79 (41.4%)	41 (34.2%)	77 (38.7%)	58 (40.3%)	19 (34.5%)	39 (37.5%)	18 (40.3%)	21 (33.3%)
	CC	8 -0,07	4 (7.7%)	4 (6.6%)	17 (5.4%)	8 (4.2%)	9 (7.5%)	9 (4.5%)	6 (4.2%)	3 (5.5%)	8 (7.7%)	2 (4.9%)	6 (9.5%)
rs533021G/A	GG	23 (23.7%)	10 (22.2%)	13 -0,25	84 (29.1%)	56 (31.5%)	28 (25.7%)	57 (30.3%)	42 (31.1%)	15 (28.3%)	26 -0,28	14 (36.8%)	12 (21.8%)
	GA	55 (56.7%)	28 (62.7%)	27 (51.9%)	136 (47.1%)	86 (48.3%)	48 (44.0%)	85 (45.2%)	63 (46.7%)	22 (41.5%)	45 (48.4%)	19 -0,5	26 (47.3%)
	AA	19 (19.6%)	7 (15.6%)	12 (23.1%)	69 (23.9%)	36 (20.2%)	33 (30.3%)	46 (24.5%)	30 (22.2%)	16 (30.2%)	22 (23.7%)	5 (13.2%)	17 (30.9%)
rs492038T/G	TT	38 (33.9%)	15 (30.6%)	23 (37.1%)	87 (28.3%)	48 (25.8%)	37 (31.1%)	59 (30.3%)	42 (30.0%)	17 (30.9%)	23 (22.3%)	4 (9.8%)	19 (30.6%)
	TG	49 (43.8%)	23 (46.9%)	25 (40.3%)	156 (50.8%)	96 (51.6%)	60 (50.4%)	94 (48.2%)	65 (47.1%)	28 (50.9%)	58 (56.3%)	27 (65.9%)	31 (50.0%)
	GG	25 (22.3%)	11 (22.4%)	14 (22.6%)	64 (20.8%)	42 (22.6%)	22 (18.5%)	42 (21.5%)	32 (22.9%)	10 (18.2%)	22 (21.4%)	10 (24.4%)	12 (19.4%)
rs4924215G/A	GG	67 (59.8%)	28 (56.0%)	39 (63.9%)	160 (51.4%)	103 (54.5%)	57 (47.5%)	102 (51.5%)	79 (55.2%)	23 (41.8%)	54 (52.4%)	20 -0,5	34 -0,54
	GA	41 (36.6%)	20 (40.0%)	20 (32.8%)	120 (38.6%)	69 (36.5%)	50 (41.7%)	79 (39.9%)	51 (35.7%)	28 (50.9%)	37 (35.9%)	17 (42.5%)	20 (31.7%)
	AA	4 (3.6%)	2 (4.0%)	2 (3.3%)	31 -0,1	17 (9.0%)	13 (10.8%)	17 (8.6%)	13 (9.1%)	4 (7.3%)	12 (11.7%)	3 (7.5%)	9 (14.3%)
rs4924214T/C	TT	56 (52.8%)	26 (58.5%)	30 (50.8%)	165 (54.6%)	94 (51.4%)	69 (59.0%)	101 (52.3%)	70 (50.7%)	31 (56.4%)	57 (56.4%)	20 -0,5	37 (60.7%)
	TC	44 (41.5%)	17 -0,37	26 (44.1%)	113 (37.4%)	74 (40.4%)	39 (33.3%)	74 (38.3%)	55 (39.9%)	19 (34.5%)	38 (37.6%)	18 (45.0%)	20 (32.8%)
	CC	6 (5.7%)	3 (6.5%)	3 (5.1%)	24 (7.9%)	15 (8.2%)	9 (7.7%)	18 (9.3%)	13 (9.4%)	5 (9.1%)	6 (5.9%)	2 (5.0%)	4 (6.6%)
rs7179902A/T	AA	80 (71.4%)	39 (76.5%)	41 (68.3%)	215 (69.4%)	136 (72.0%)	78 (65.5%)	138 (70.1%)	104 (73.2%)	34 (61.8%)	71 (68.9%)	28 (68.3%)	43 (69.4%)
	AT	29 (25.9%)	11 (21.6%)	17 (28.3%)	80 (25.8%)	45 (23.8%)	34 (28.6%)	50 (25.4%)	32 (22.5%)	18 (32.7%)	26 (25.2%)	11 (26.8%)	15 (24.2%)
	TT	3 (2.7%)	1 (2.0%)	2 (3.3%)	15 (4.8%)	8 (4.2%)	7 (5.9%)	9 (4.5%)	6 (4.2%)	3 (5.5%)	6 (5.8%)	2 (4.9%)	4 (6.5%)

The rs1851317 marker was not informative in our study population.

Association studies were done between CCC and ASY (Table 3) or between CCC with a left ventricular ejection fraction value under 0.4% and ASY (Table 4). Among the asymptomatic individuals (used here as controls), 31.8% carried the rs640249CC genotype, 43.4% carried the rs640249CA genotype and 24.8% carried the rs640249AA genotype. In the CCC patient group, 37.7% of the subjects carried the rs640249CC genotype, 49.2% carried the rs640249CA genotype and 13.1% carried the rs640249AA genotype. 

**Table 3:** Association studies between CCC and ASY including as covariates the gender and the polymorphism one by one.

**Tag SNP**		**Genotype groups**	**Association test**
**rs639735**	**C/T**	**CC vs CT+TT**	**p=0.435; OR(95%CI)=1.10(0.88-1.36)**
		CC vs CT	p=0.598; OR(95%CI)=1.13(0.71-1.80)
		CC vs TT	p=0.328; OR(95%CI)=1.20(0.83-1.74)
**rs640249**	**C/A**	**AA vs CA+CC**	**p=0.006; OR(95%CI)=1.47(1.11-1.93)**
		AAvsCA	p=0.018; OR(95%CI)=2.04(1.13-3.66)
		AAvsCC	p=0.008; OR(95%CI)=1.52(1.11-2.07)
**rs893130**	**A/T**	**AA vs AT+TT**	**p=0.426; OR(95%CI)=1.11(0.86-1.43)**
		AAvsAT	p=0.526; OR(95%CI)=1.19(0.70-2.01)
		AAvsTT	p=0.449; OR(95%CI)=1.36(0.61-3.01)
**rs475786**	**C/G**	**CC vs CG+GG**	**p=0.061; OR(95%CI)=1.82(0.97-3.41)**
		CCvsCG	p=0.067; OR(95%CI)=1.80(0.96-3.37)
**rs893131**	**T/C**	**TT vs TC+CC**	**p=0.246; OR(95%CI)=1.35(0.83-2.08)**
		TTvsTC	p=0.459; OR(95%CI)=1.20(0.75-1.93)
		TTvsCC	p=0.136; OR(95%CI)=1.61(0.86-3.03)
**rs893132**	**T/C**	**TT vs TC+CC**	**p=0.234; OR(95%CI)=1.31(0.84-2.05)**
		TTvsTC	p=0.420; OR(95%CI)=1.21(0.76-1.94)
		TTvsCC	p=0.100; OR(95%CI)=1.49(0.93-2.39)
**rs533225**	**A/T**	**AA vs AT+TT**	**p=0.459; OR(95%CI)=1.29(0.66-1.52)**
		AAvsAT	p=0.536; OR(95%CI)=1.25(0.62-2.49)
		AAvsTT	p=0.569; OR(95%CI)=1.39(0.44-4.39)
**rs670957**	**G/A**	**GG vs GA+AA**	**p=0.217; OR(95%CI)=1.16(0.91-1.48)**
		GGvsGA	p=0.297; OR(95%CI)=1.31(0.79-2.20)
		GGvsAA	p=0.251; OR(95%CI)=1.20(0.88-1.62)
**rs525720**	**G/A**	**GG vs GA+AA**	**p=0.400; OR(95%CI)=1.29(0.72-2.31)**
		GGvsGA	p=0.641; OR(95%CI)=1.16(0.63-2.13)
		GGvsAA	p=0.100; OR(95%CI)=2.00(0.88-4.54)
**rs7166484**	**G/A**	**GG vs GA+AA**	**p=0.273; OR(95%CI)=1.13(0.90-1.43)**
		GGvsGA	p=0.403; OR(95%CI)=1.15(0.83-1.60)
		GGvsAA	p=0.313; OR(95%CI)=1.28(0.79-2.09)
**rs2070664**	**A/G**	**AA vs AG+GG**	**p=0.401; OR(95%CI)=1.10(0.88-1.38)**
		AAvsAG	p=0.226; OR(95%CI)=1.34(0.84-2.14)
		AAvsGG	p=0.529; OR(95%CI)=1.13(0.77-1.66)
**rs3729755**	**G/C**	**GG vs GC+CC**	**p=0.326; OR(95%CI)=1.12(0.90-1.40)**
		GGvsGC	p=0.795; OR(95%CI)=1.06(0.68-1.66)
		GGvsGC	p=0.240; OR(95%CI)=1.33(0.83-2.13)
**rs533021**	**G/A**	**GG vs GA+AA**	**p=0.345; OR(95%CI)=1.14(0.87-1.49)**
		GGvsGA	p=0.160; OR(95%CI)=1.50(0.85-2.63)
		GGvsAA	p=0.779; OR(95%CI)=1.05(0.74-1.49)
**rs492038**	**T/G**	**TT vs TG+GG**	**p=0.291; OR(95%CI)=1.29(0.80-2.07)**
		TTvsTG	p=0.193; OR(95%CI)=1.40(0.84-2.32)
		TTvsGG	p=0.815; OR(95%CI)=1.04(0.76-1.41)
**rs4924215**	**G/A**	**GG vs GA+AA**	**p=0.105; OR(95%CI)=1.22(0.96-1.50)**
		GGvsGA	p=0.316; OR(95%CI)=1.27(0.80-2.02)
		GGvsAA	p=0.032; OR(95%CI)=1.82(1.05-3.14)
**rs4924214**	**T/C**	**TT vs TC+CC**	**p=0.766; OR(95%CI)=1.07(0.68-1.68)**
		TTvsTC	p=0.586; OR(95%CI)=1.14(0.71-1.82)
		TTvsCC	p=0.540; OR(95%CI)=1.16(0.72-1.88)
**rs7179902**	**A/T**	**AA vs AT+TT**	**p=0.799; OR(95%CI)=1.07(0.65-1.77)**
		AAvsAT	p=0.697; OR(95%CI)=1.10(0.67-1.84)
		AAvsTT	p=0.286; OR(95%CI)=1.42(0.75-2.68)

**Table 4:** Association studies between CCC with a left ventricular ejection fraction value under 0.4% and ASY including as covariates the gender and the polymorphism one by one.

**Tag SNP**		**Genotype groups**	**Association test**
**rs639735**	**C/T**	**CC vs CT+TT**	**p=0.287; OR(95%CI)=1.14(0.90-1.45)**
		CC vs CT	p=0.405; OR(95%CI)=1.24(0.75-2.07)
		CC vs TT	p=0.292; OR(95%CI)=1.26(0.82-1.92)
**rs640249**	**C/A**	**AA vs CA+CC**	**p=0.003; OR(95%CI)=1.61(1.17-2.21)**
		AAvsCA	p=0.011; OR(95%CI)=2.38(1.22-4.65)
		AAvsCC	p=0.004; OR(95%CI)=1.70(1.19-2.44)
**rs893130**	**A/T**	**AA vs AT+TT**	**p=0.401; OR(95%CI)=1.13(0.85-1.49)**
		AAvsAT	p=0.473; OR(95%CI)=2.70(0.69-2.20)
		AAvsTT	p=0.523; OR(95%CI)=1.33(0.55-3.19)
**rs475786**	**C/G**	**CC vs CG+GG**	**p=0.062; OR(95%CI)=1.92(0.97-3.79)**
		CCvsCG	p=0.071; OR(95%CI)=1.88(0.95-3.73)
**rs893131**	**T/C**	**TT vs TC+CC**	**p=0.540; OR(95%CI)=1.17(0.71-1.95)**
		TTvsTC	p=0.806; OR(95%CI)=1.07(0.63-1.81)
		TTvsCC	p=0.212; OR(95%CI)=1.53(0.78-3.00)
**rs893132**	**T/C**	**TT vs TC+CC**	**p=0.163; OR(95%CI)=1.42(0.87-2.34)**
		TTvsTC	p=0.343; OR(95%CI)=1.29(0.77-2.16)
		TTvsCC	p=0.063; OR(95%CI)=1.66(0.97-2.85)
**rs533225**	**A/T**	**AA vs AT+TT**	**p=0.774; OR(95%CI)=1.12(0.52-2.38)**
		AAvsAT	p=0.942; OR(95%CI)=1.03(0.47-2.25)
		AAvsTT	p=0.456; OR(95%CI)=1.59(0.47-5.38)
**rs670957**	**G/A**	**GG vs GA+AA**	**p=0.087; OR(95%CI)=1.27(0.97-1.66)**
		GGvsGA	p=0.087; OR(95%CI)=1.65(0.93-2.93)
		GGvsAA	p=0.268; OR(95%CI)=1.22(0.86-1.72)
**rs525720**	**G/A**	**GG vs GA+AA**	**p=0.613; OR(95%CI)=1.18(0.62-2.23)**
		GGvsGA	p=0.904; OR(95%CI)=1.04(0.54-2.02)
		GGvsAA	p=0.107; OR(95%CI)=2.61(0.81-8.35)
**rs7166484**	**G/A**	**GG vs GA+AA**	**p=0.068; OR(95%CI)=1.27(0.98-1.64)**
		GGvsGA	p=0.247; OR(95%CI)=1.24(0.86-1.77)
		GGvsAA	p=0.069; OR(95%CI)=1.65(0.96-2.84)
**rs2070664**	**A/G**	**AA vs AG+GG**	**p=0.766; OR(95%CI)=1.04(0.81-1.34)**
		AAvsAG	p=0.964; OR(95%CI)=1.01(0.60-1.70)
		AAvsGG	p=0.239; OR(95%CI)=1.29(0.84-1.97)
**rs3729755**	**G/C**	**GG vs GC+CC**	**p=0.504; OR(95%CI)=1.09(0.85-1.39)**
		GGvsGC	p=0.488; OR(95%CI)=1.20(0.72-2.01)
		GGvsGC	p=0.334; OR(95%CI)=1.29(0.77-2.16)
**rs533021**	**G/A**	**GG vs GA+AA**	**p=0.040; OR(95%CI)=1.31(1.01-1.69)**
		GGvsGA	p=0.106; OR(95%CI)=1.66(0.90-3.06)
		GGvsAA	p=0.828; OR(95%CI)=1.04(0.72-1.52)
**rs492038**	**T/G**	**TT vs TG+GG**	**p=0.591; OR(95%CI)=1.15(0.69-1.93)**
		TTvsTG	p=0.474; OR(95%CI)=1.22(0.70-2.13)
		TTvsGG	p=0.985; OR(95%CI)=1.00(0.72-1.41)
**rs4924215**	**G/A**	**GG vs GA+AA**	**p=0.100; OR(95%CI)=1.23(0.96-1.57)**
		GGvsGA	p=0.209; OR(95%CI)=1.39(0.83-2.31)
		GGvsAA	p=0.096; OR(95%CI)=1.66(0.91-3.00)
**rs4924214**	**T/C**	**TT vs TC+CC**	**p=0.914; OR(95%CI)=1.03(0.63-1.69)**
		TTvsTC	p=0.833; OR(95%CI)=1.06(0.63-1.78)
		TTvsCC	p=0.351; OR(95%CI)=1.27(0.77-2.10)
**rs7179902**	**A/T**	**AA vs AT+TT**	**p=0.647; OR(95%CI)=1.14(0.65-1.99)**
		AAvsAT	p=0.568; OR(95%CI)=1.18(0.67-2.07)
		AAvsTT	p=0.330; OR(95%CI)=1.41(0.71-2.82)

For the rs640249 polymorphism, we compared subjects carrying the AA genotype with those carrying either the CC genotype or the CA genotype. We included sex in the analysis as a covariate. The two covariates remained significantly associated with disease (sex: *p*=0.006; OR(95%CI)=1.86(1.19-2.89); rs640249: *p*=0.006; OR(95%CI)=1.47(1.11-1.93); Table 3). We then restricted the analysis to patients with severe ventricular dysfunction (left ventricular ejection fraction < 40%). In this subgroup, 40.0% of the subjects carried the rs640249CC genotype, 48.2% of the subjects carried the rs640249CA genotype and 11.8% of the subjects carried the rs640249AA genotype. In multivariate binary regression analysis, sex and the rs640249 polymorphism remained significantly associated with CCC (sex: *p*=8.6 x 10^-6^; OR(95%CI)=3.08(1.87-5.04); rs640249: *p*=0.003; OR(95%CI)=1.61(1.17-2.21); Table 4). This polymorphism did not distinguish moderate CCC from severe CCC and moderate CCC from ASY controls.

 We performed the same analyses on male subjects only (193 cases vs 53 ASY), as the risk of progression to CCC is higher in men than in women. In comparisons of asymptomatic subjects and CCC patients, the rs640249 polymorphism was found to be significantly associated with CCC (*p*=0.008; OR(95%CI)=1.67(1.14-2.43)). This association was also significant in comparisons of severe CCC patients with asymptomatic subjects (*p*=0.015; OR(95%CI)=1.64(1.10-2.44)). Thus, the rs640249CC genotype is significantly associated with a higher risk of developing CCC.

**Figure 2:**
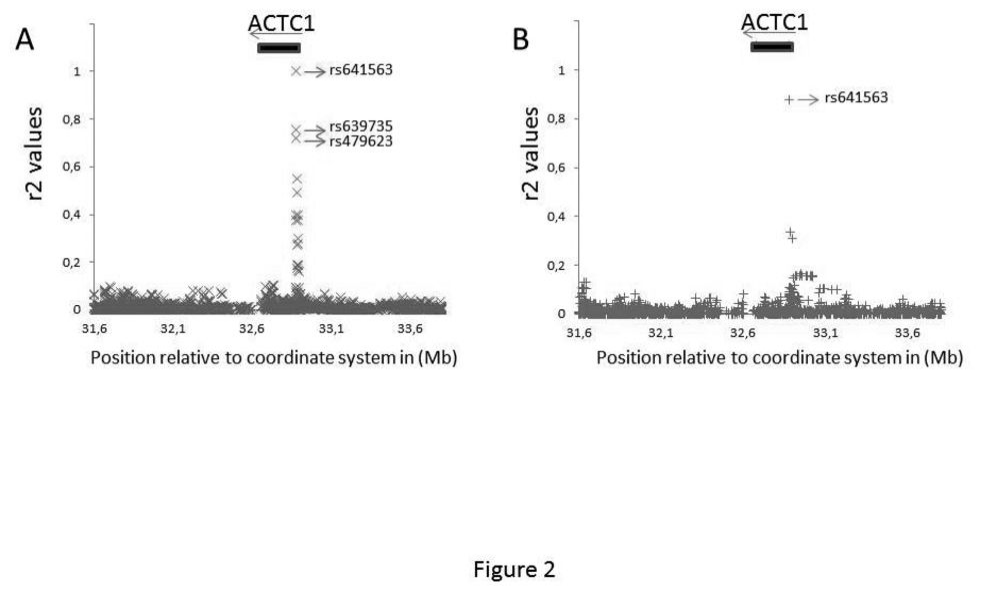
Correlation analysis for the rs640249 polymorphism in two different reference populations. Genotype data from the European reference population (CEU) and from the West Africa reference population (YRI) were downloaded from HapMap. A 2 Mb region surrounding the ACTC1 gene was analyzed in these two populations. The data were analyzed with Haploview Software. **A)** Correlations were assessed by calculating r^2^ values. The rs640249 polymorphism was found to be correlated with three other polymorphisms in the CEU reference population (rs641563; rs639735; rs479623). **B**) In the West Africa reference population, the rs640249 polymorphism was correlated only with rs641563.

We performed a linkage disequilibrium analysis on the two reference populations. We focused this analysis on a 2 Mb region surrounding the rs640249 polymorphism Figure 2. In the CEU reference population, the rs640249 polymorphism was found to be correlated with three other polymorphisms located in the *ACTC1* gene promoter (rs641563: r^2^=1; rs639735: r^2^=0.76; rs479623: r^2^=0.72; Figure 2A). In the Yoruba reference population, the rs640249 polymorphism was correlated only with the rs641563 polymorphism (r^2^=0.88) (Figure 2B). These three markers are all within 1.5 Kb of the rs640249 polymorphism. Among the asymptomatic individuals, 17.8% of the subjects carried the rs479623CC genotype, 50.5% carried the rs479623TC genotype and 31.8% carried the rs479623TT genotype. In the patient group, 14.1% of the subjects carried the rs479623CC genotype, 51.7% carried the rs479623TC genotype and 34.2% carried the rs479623TT genotype. In the patient group, 17.8% of the subjects carried the rs479623CC genotype, 50.5% carried the rs479623TC genotype and 31.8% carried the rs479623TT genotype. For the rs641563 polymorphism, we compared the subjects carrying the AA genotype with the others. Some trends for association were detected (sex: *p*=0.002; OR(95%CI)=2.11(1.33-3.36); rs641563: *p*=0.078; OR(95%CI)=1.67(0.94-2.97)). Similar results were obtained when we compared severe CCC to ASY (sex: *p*=6.1 x 10^-6^; OR(95%CI)=3.28(1.96-5.49); rs641563: *p*=0.054; OR(95%CI)=1.90(0.99-3.66)). 

**Figure 3:**
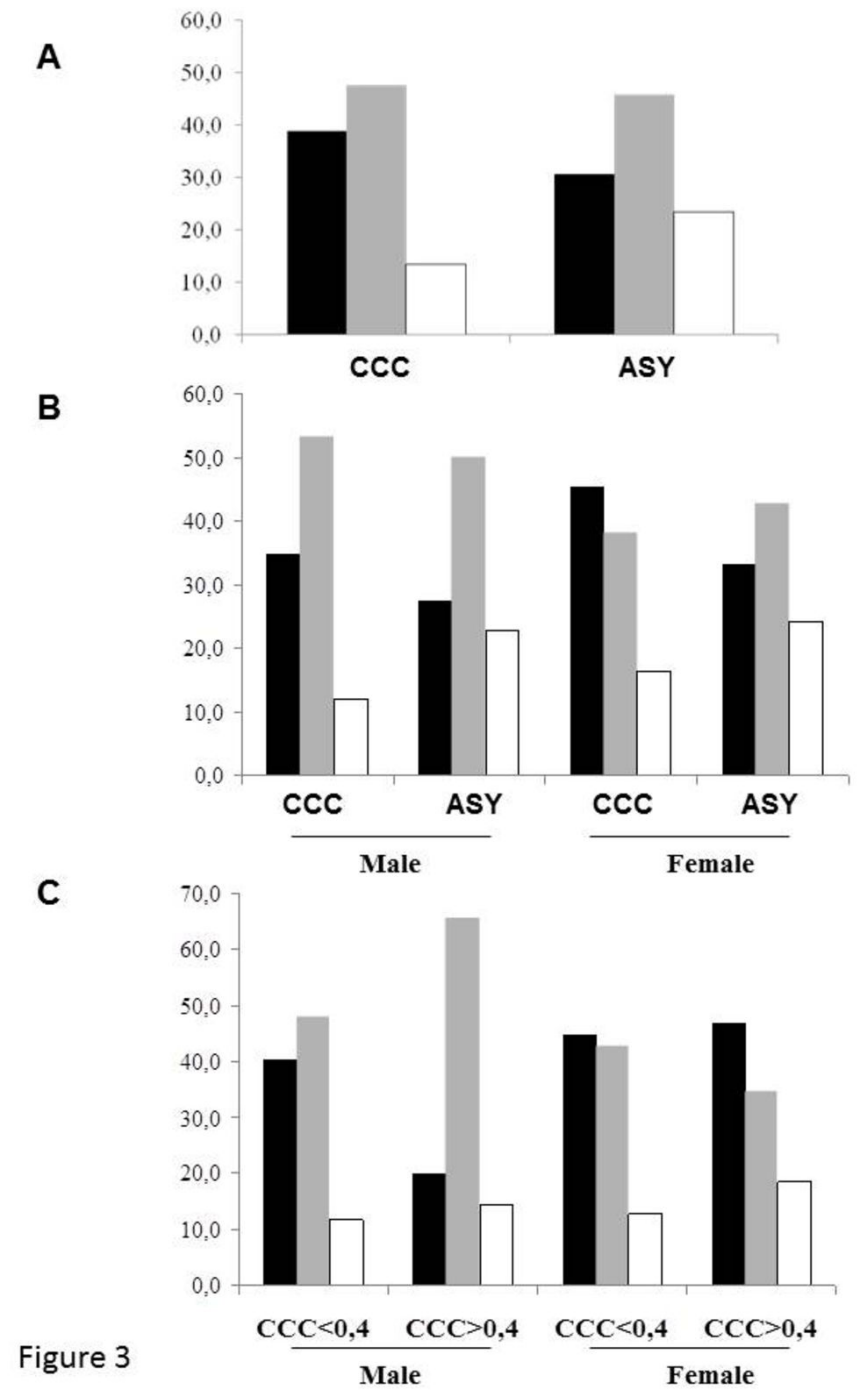
The rs640249A-rs641563A haplotype is associated with resistance to CCC. Based on the genotypes obtained, we performed a haplotype analysis of the rs7719175 and rs1800925 polymorphisms. Only three haplotypes were found in our study population: **A**) Distribution of the three main haplotype combinations in the CCC patients and ASY subjects: homozygous rs640249C-rs641563C (black bar); heterozygous rs640249C-rs641563C + rs640249A-rs641563A (gray bar) and homozygous rs640249A-rs641563A (white bar). **B**) Haplotype combinations between cases and controls, taking sex into account. **C**) Distribution of the three main haplotype combinations between patients with severe and moderate CCC, taking sex into account.

We performed a haplotype reconstruction of the study population based on the SNPs rs640249 and rs641563. Four haplotypes were detected with various frequencies: (1) rs640249**C**-rs641563**C**: 58.8%; (2) rs640249**A**-rs641563**A**: 39.1%; (3) rs640249**C**-rs641563**A**: 1.6%; (4) rs640249**A**-rs641563**C**: 0.5%. We investigated the relationship between Chagas disease and the two most frequent haplotypes (Figure 3). In a multivariate analysis, including sex and haplotype combination as covariates, comparing CCC patients with asymptomatic subjects, some significant associations were detected (sex: *p*=0.005; OR(95%CI)=1.99(1.24-3.20); haplotype combination: *p*=0.025; OR(95%CI)=1.21(1.02-1.43)) (Figure 3). The rs640249**A**-rs641563**A** haplotype was found to be associated with a lower risk of developing CCC. This haplotype was also shown to be associated with a lower risk of developing CCC in severe cases (sex: *p*=2.5 x 10^-5^; OR(95%CI)=3.12(1.84-5.30); haplotype combinations: *p*=0.012; OR(95%CI)=1.27(1.05-1.54)) (Figure 3). The authors had access to a limited number of tissue samples extracted from CCC patients. Thirty three percent were homozygous for the susceptibility haplotype (rs640249**C**-rs641563**C**), 67% were heterozygous and none of them were homozygous for the protective haplotype (rs640249**A**-rs641563**A**).

**Figure 4:**
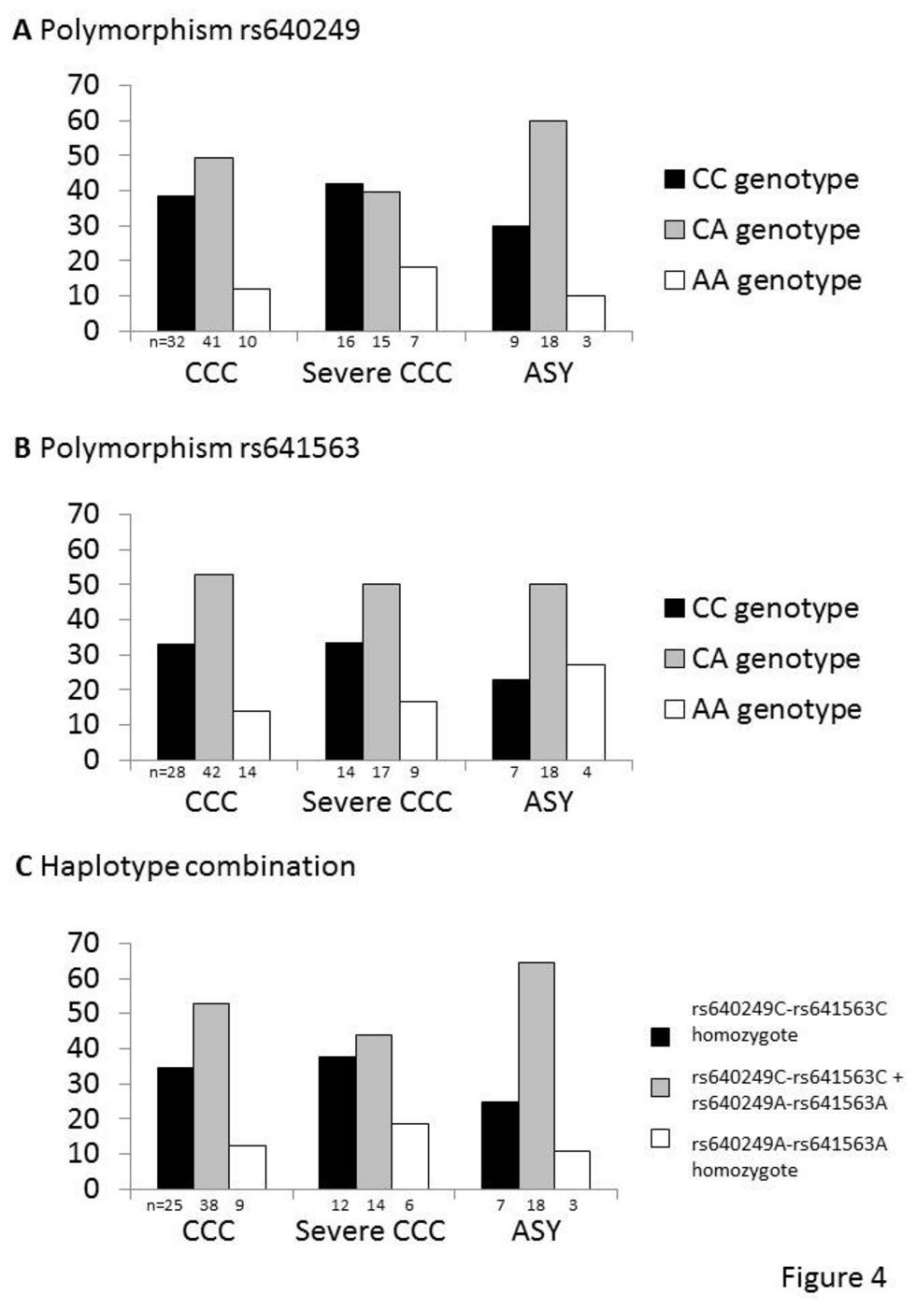
The same trends for association were detected in a small, independent replication cohort. The two main polymorphisms, rs640249 and rs641563, were genotyped in the independent replication cohort. This second cohort focused exclusively on male patients with Chagas disease, who have a higher risk of progression to CCC. This replication cohort included asymptomatic (*n* = 36) and CCC patients (*n* = 102). Of the 106 patients with CCC, 48 had severe ventricular dysfunction (left ventricular ejection fraction <40%). The rs640249 genotype distribution is illustrated in A. The results for the rs641563 polymorphism are in B. Haplotype analysis was then performed in C.

We enrolled a second independent cohort, including only male subjects, who have a higher risk of progression to CCC. We included 102 cases of CCC, 48 (47%) of whom developed severe chronic cardiomyopathy and 36 asymptomatic subjects. For the rs640249 polymorphism, the susceptibility genotype was carried by 38.6% of the CCC patients, 42.1% of the severe CCC patients and only 30% of the asymptomatic subjects (Figure 4). Similarly, 33.3% of the CCC patients and 35% of the severe CCC patients carried the rs641563 susceptibility genotype whereas 24.1% of the asymptomatic subjects carried this genotype (Figure 4). A haplotype analysis was also performed on this new cohort. Overall, 34.7% of the CCC patients and 37.5% of the severe CCC patients were homozygous for the susceptibility haplotype, whereas only 25% of the asymptomatic subjects carried this haplotype combination (Figure 4). However, although these results confirmed those obtained with the original cohort, the significance threshold for association was not reached, probably due to the size of the replication cohort.

**Figure 5:**
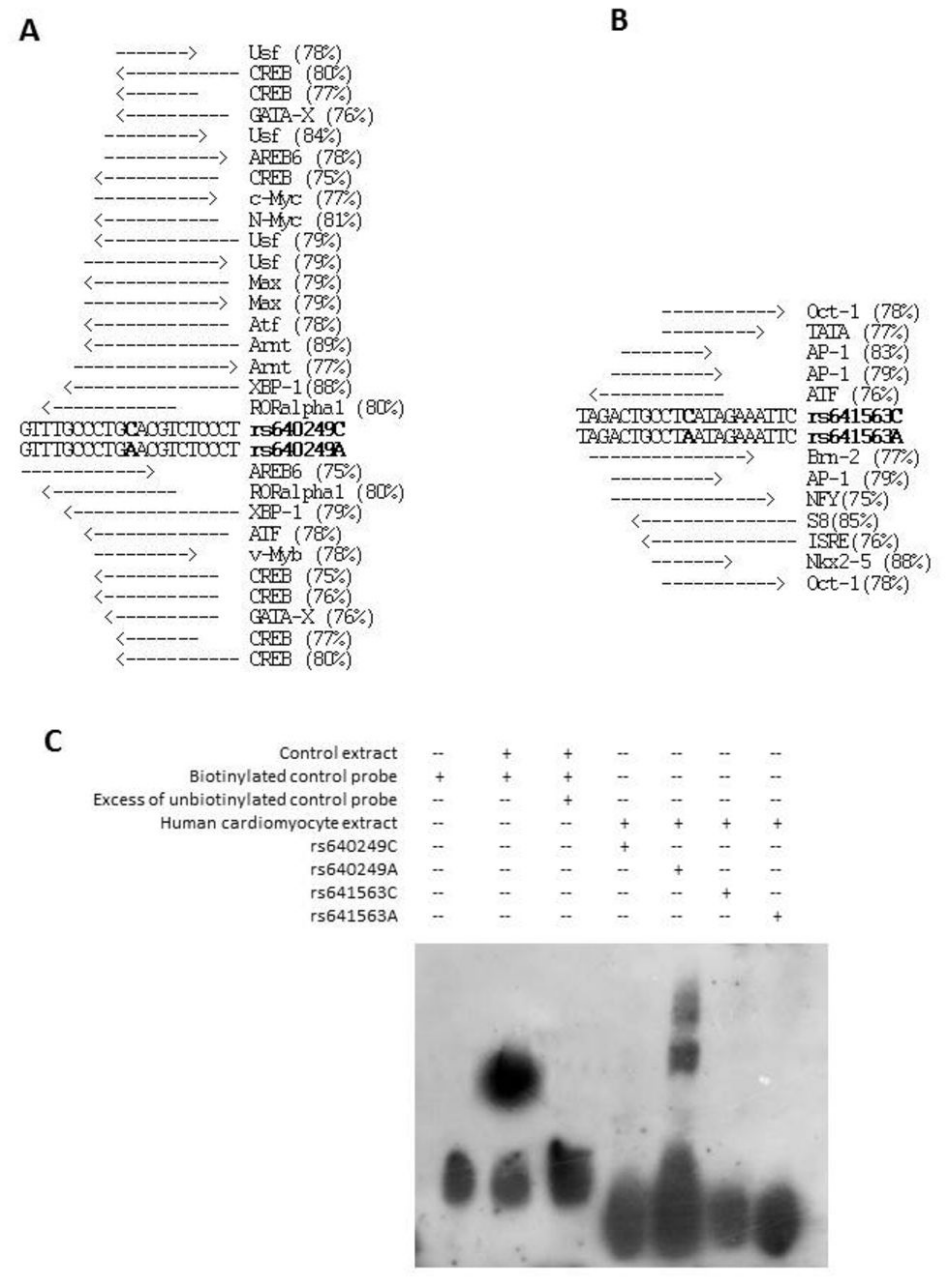
*In silico* analysis predicted differential binding patterns for rs640249 and rs641563 polymorphisms. Gel shift experiment has confirmed this differential binding for rs640249 polymorphism. The probability of these polymorphisms creating or altering DNA–protein interaction was determined by in silico analysis (http://www.gene-regulation.com/cgi-bin/pub/programs/match/bin/match.cgi). An 75% threshold score (similarity matrix) was used. For each polymorphism (A, rs1800925; B, rs641563) putative bindings are described. The similarity matrix score is indicated in brackets. **C**: Electrophoretic mobility shift assays were performed in vitro as described in Materials and Methods. A differential binding pattern was detected for the rs640249A allele.

The rs640249 and rs641563 polymorphisms are located in the promoter region. Sequence changes can influence gene expression through the creation or alteration of DNA-binding sites for transcription factors. We performed an *in silico* analysis of these polymorphic loci for potential differences in transcription factor-binding sites (Figure 5). We performed an Electrophoretic mobility shift assay (EMSA) using nuclear extracts from primary human cardiomyocyte culture without ex vivo additional stimulation. We showed that the protective rs640249A allele binds some transcription factors that fail to bind to the rs640249C allele (Figure 5). No differential binding pattern was detected for the rs641563 polymorphism.

## Discussion

So far, the search for associations between candidate genes with the development of CCC has been focused mainly on genes involved in immune response and inflammation [27]. However, CCC is not a monogenic disease, and it is likely that the progression to overt inflammatory dilated cardiomyopathy may result from the combined effect and inadequate counter regulation of relevant genes. In spite of the important role of direct inflammatory damage, genes involved in the resilience of myocardial tissue to inflammatory and other kinds of stress could potentially also be involved. 

We investigated the hypothesis that the *ACTC1* gene plays an essential role in the development of CCC. A lower level of ACTC1 protein was detected in myocardial tissue from patients with severe CCC than in such tissue from uninfected subjects. Gene expression analysis has also shown the *ACTC1* gene to be under expressed in the myocardium of CCC patients, although not to a statistically significant extent. Our finding that the myocardium of CCC patients contains lower ACTC1 protein than control myocardial tissue is consistent with the findings of Jiang et al. (2010) for congenital heart disease. They found that low levels of *ACTC1* mRNA and protein were strongly correlated with cardiomyocyte apoptosis, as assessed by TUNEL staining [33]. Tostes et al. demonstrated the occurrence of significantly higher than normal levels of cardiomyocyte apoptosis in myocardial tissue from severe CCC cases [34]. The finding that a forced reduction of *ACTC1* expression in cardiomyocytes leads to higher levels of apoptosis [33] and consequently cardiomyocyte loss [35] indicates a possible explanation for the involvement of reduced ACTC1 protein levels in ventricular dysfunction in CCC. Although the possible mechanisms involved in actin deficiency-induced cardiomyocyte apoptosis are still unknown, actin:myosin stochiometry imbalance is one possible mechanism whereby reduced actin levels would lead to cardiomyocyte apoptosis and, possibly, reduced ventricular function. Beall et al. (1989) have shown that actin deficiency and the resulting stoichiometry imbalance can significantly affect proper sarcomere formation with ensuing functional implications in striated muscle in the *Drosophila* model [48]. It has recently been reported that alpha-cardiac actin binds to titin and telethonin, along with several other signaling proteins in the Z-disc region of the sarcomere, which is now known to be the sarcomeric mechanosensor, responsible for downstream signal transduction [49]. It has been shown in murine models that the Z-disc actin-binding protein telethonin links mechanical and other sarcomere stress with cardiomyocyte apoptosis via direct interaction with the propapoptotic p53 protein [50]. It is conceivable that reduced levels of alpha-cardiac actin could impact on Z-disc mechanosensor function and thereby enhance cardiomyocyte apoptosis. 

We investigated the possibility that polymorphisms in or around the *ACTC1* gene control susceptibility to CCC. We found a significant association between the rs640249 polymorphism and CCC, which was confirmed when we compared severe CCC patients with ASY controls. The rs640249C allele was found to be associated with susceptibility to CCC, whereas the rs640249A allele was associated with resistance to CCC development. This is the first polymorphism in a gene encoding a structural heart protein to be associated with progression of Chronic Chagas disease, implying that genetically determined heart tissue fragility may play a role in pathogenesis and development of CCC. Moreover, the rs640249 polymorphism was correlated with three neighboring polymorphisms (rs641563, rs639735 and rs479623). Only one of these markers (rs641563) was also associated with CCC development. This marker, like rs640249, could not distinguish between moderate and severe cases of CCC. A haplotype analysis, based on the genotype of these two markers, led to the identification of a susceptibility haplotype (rs640249**C**-rs641563**C**) and a protective haplotype (rs640249**A**-rs641563**A**). 

The results presented here are not corrected for multiple comparisons. We performed correction according to Nyholt’s procedure [45,46]. Only the rs640219 polymorphism remains significantly associated on the main cohort and this one seems to be functional. Indeed, our results with the EMSA assay indicate that the rs640249 polymorphism affects the binding of regulatory elements, such as transcriptional factors, in the promoter regions of the *ACTC1* gene. Our *in silico* analysis indicate some candidate transcription factors that could potentially bind differentially to the sequences of the two allelic forms. Given the importance of ACTC1 expression for proper cardiac muscle function, the rs640249 polymorphism may contribute to disease severity. The protein level decrease (-64 %) in the myocardium of CCC patients than in myocardial samples from individuals without cardiomyopathies is completely in agreement with the available data obtained on acute in syrian hamster model [36]. 

However, the association test results remain borderline after correction for multiple testing. The same trend of association was detected on our independent replication cohort. The p values that we obtained are modest either by the fact that the size of our cohort is limited (even if it is one of the largest described so far) or by the fact that the impact of the polymorphism on the development of severe cardiomyopathy is limited. However, it is important to notice that the proteomic analysis, genetic association tests, haplotype analysis and EMSA experiment data are going in the same way. Authors think that it will be better to confirm these results in another large independent cohort or in other etiologies of myocarditis and inflammatory cardiomyopathy, such as viral myocarditis and post-viral cardiomyopathy.

To our knowledge, this is the first demonstration of a putative association between polymorphisms in a non-inflammatory/immune response gene – and in this case, a structural cardiac sarcomeric protein-encoding gene and susceptibility to CCC. Proinflammatory cytokines that are found to be upregulated in plasma and heart tissue of heart failure [51] and CCC patients [52] can promote cardiomyocyte apoptosis. Since reduced transcription levels of cardiac actin have been associated with increased susceptibility to apoptosis, it is conceivable that increased levels of inflammatory cytokines, secondary to environmental or genetic causes could differentially affect cardiomyocyte function in carriers of either allelic form of the ACTC1 gene. Hypothetically, the interaction of polymorphisms controlling inflammatory cytokines with that of cardiomyocyte alpha-cardiac actin could act synergistically, leading to different levels if cardiomyocyte apoptosis among chronic Chagas cardiomyopathy patients. Thus, in addition to inflammation, genetically determined myocardial resilience may be an important factor for progression to CCC. In this analysis, the odd ratio values for the associated markers remain modest similar to the previous candidate gene analyses. Increase sample sizes will permit to test all the associated genes on the same population and define the relative contribution of each gene. 

Taken together, these reports may suggest that variations in the expression of the *ACTC1* gene may play an important role in the pathogenesis of Chagas disease cardiomyopathy. 

## Supporting Information

Figure S1 **Relative quantification of alpha-cardiac actin 1 (ACTC1) by immunoblotting (duplicate analysis).** Myocardial samples were obtained from the left ventricular free wall of the hearts of patients with severe CCC and end-stage heart failure, at the time of heart transplantation. Samples from five hearts from CCC patients (at least two positive results in three independent anti-*T*. *cruzi* serology tests, as indicated above), and from healthy hearts from organ donors not used for transplantation for technical reasons were used. Immunoblotting and protein quantification were done in duplicate. The immunoblot and the protein quantification result of the second experiment are presented here. The central line represents the median. Representative results from two experiments are shown here. A Mann-Whitney test was performed and differences were considered significant if *P*<0.001.
(TIF)

Table S1 **Characteristics of heart sample donors.** EF: Ejection Fraction (reference value: ≥55%), LVDD: *Left* *Ventricular* *Diastolic* *Diameter* (reference value: 39-53mm). N: individuals without cardiomyopathies, CCC: chronic Chagas disease cardiomyopathy. nd: not done.
(DOCX)

## References

[1] Salvatella R (2007) Andean subregional Chagas disease area and the Andean initiative of Chagas disease. Mem Inst Oswaldo Cruz 102 Suppl 1: 39-40. doi:10.1590/S0074-02762007005000105. PubMed: 17906804. 17906804

[2] Kirchhoff LV, Weiss LM, Wittner M, Tanowitz HB (2004) Parasitic diseases of the heart. Front Biosci 9: 706-723. doi:10.2741/1255. PubMed: 14766402. 14766402

[3] Schofield CJ, Jannin J, Salvatella R (2006) The future of Chagas disease control. Trends Parasitol 22: 583-588. doi:10.1016/j.pt.2006.09.011. PubMed: 17049308. 17049308

[4] Dias E, Laranja FS, Miranda A, Nobrega G (1956) Chagas' disease; a clinical, epidemiologic, and pathologic study. Circulation 14: 1035-1060. doi:10.1161/01.CIR.14.6.1035. PubMed: 13383798. 13383798

[5] Martin UO, Afchain D, de Marteleur A, Ledesma O, Capron A (1987) Circulating immune complexes in different developmental stages of Chagas' disease. Medicina (B Aires) 47: 159-162. 3121979

[6] Coura JR (2007) Chagas disease: what is known and what is needed--a background article. Mem Inst Oswaldo Cruz 102 Suppl 1: 113-122. PubMed: 17992371. 17992371 10.1590/s0074-02762007000900018

[7] Mady C, Cardoso RH, Barretto AC, da Luz PL, Bellotti G et al. (1994) Survival and predictors of survival in patients with congestive heart failure due to Chagas' cardiomyopathy. Circulation 90: 3098-3102. doi:10.1161/01.CIR.90.6.3098. PubMed: 7994859. 7994859

[8] Bestetti RB, Muccillo G (1997) Clinical course of Chagas' heart disease: a comparison with dilated cardiomyopathy. Int J Cardiol 60: 187-193. doi:10.1016/S0167-5273(97)00083-1. PubMed: 9226290. 9226290

[9] Pereira Barretto AC, Mady C, Arteaga-Fernandez E, Stolf N, Lopes EA et al. (1986) Right ventricular endomyocardial biopsy in chronic Chagas' disease. Am Heart J 111: 307-312. doi:10.1016/0002-8703(86)90144-4. PubMed: 3946173. 3946173

[10] Higuchi ML, De Morais CF, Pereira Barreto AC, Lopes EA, Stolf N et al. (1987) The role of active myocarditis in the development of heart failure in chronic Chagas' disease: a study based on endomyocardial biopsies. Clin Cardiol 10: 665-670. doi:10.1002/clc.4960101113. PubMed: 3677499. 3677499

[11] Ribeirao M, Pereira-Chioccola VL, Renia L, Fragata Filho Augusto A, Schenkman S et al. (2000) Chagasic patients develop a type 1 immune response to Trypanosoma cruzi trans-sialidase. Parasite Immunol 22: 49-53. doi:10.1046/j.1365-3024.2000.00260.x. PubMed: 10607290. Available online at: 10.1046/j.1365-3024.2000.00260.x Available online at: PubMed: 10607290 10607290

[12] Abel LC, Rizzo LV, Ianni B, Albuquerque F, Bacal F et al. (2001) Chronic Chagas' disease cardiomyopathy patients display an increased IFN-gamma response to Trypanosoma cruzi infection. J Autoimmun 17: 99-107. doi:10.1006/jaut.2001.0523. PubMed: 11488642. 11488642

[13] Teixeira MM, Gazzinelli RT, Silva JS (2002) Chemokines, inflammation and Trypanosoma cruzi infection. Trends Parasitol 18: 262-265. doi:10.1016/S1471-4922(02)02283-3. PubMed: 12036740. 12036740

[14] Ferreira RC, Ianni BM, Abel LC, Buck P, Mady C, et al. (2003) Increased plasma levels of tumor necrosis factor-alpha in asymptomatic/"indeterminate" and Chagas disease cardiomyopathy patients. Mem Inst Oswaldo Cruz 98: 407-11. 12886425 10.1590/s0074-02762003000300021

[15] Talvani A, Rocha MO, Barcelos LS, Gomes YM, Ribeiro AL et al. (2004) Elevated concentrations of CCL2 and tumor necrosis factor-alpha in chagasic cardiomyopathy. Clin Infect Dis 38: 943-950. doi:10.1086/381892. PubMed: 15034825. 15034825

[16] Nogueira LG, Santos RH, Ianni BM, Fiorelli AI, Mairena EC et al. (2012) Myocardial Chemokine Expression and Intensity of Myocarditis in Chagas Cardiomyopathy Are Controlled by Polymorphisms in CXCL9 and CXCL10. PLoS Negl Trop. Drosophila Inf Service 6: e1867. 10.1371/journal.pntd.0001867 PMC3493616 23150742

[17] Cunha-Neto E, Dzau VJ, Allen PD, Stamatiou D, Benvenutti L et al. (2005) Cardiac gene expression profiling provides evidence for cytokinopathy as a molecular mechanism in Chagas' disease cardiomyopathy. Am J Pathol 167: 305-313. doi:10.1016/S0002-9440(10)62976-8. PubMed: 16049318. 16049318 PMC1603558

[18] Riol-Blanco L, Sánchez-Sánchez N, Torres A, Tejedor A, Narumiya S et al. (2005) The chemokine receptor CCR7 activates in dendritic cells two signaling modules that independently regulate chemotaxis and migratory speed. J Immunol 174: 4070-4080. PubMed: 15778365. 15778365 10.4049/jimmunol.174.7.4070

[19] Sakai N, Wada T, Yokoyama H, Lipp M, Ueha S et al. (2006) Secondary lymphoid tissue chemokine (SLC/CCL21)/CCR7 signaling regulates fibrocytes in renal fibrosis. Proc Natl Acad Sci U S A 103: 14098-14103. doi:10.1073/pnas.0511200103. PubMed: 16966615. 16966615 PMC1599918

[20] Reddy VS, Harskamp RE, van Ginkel MW, Calhoon J, Baisden CE et al. (2008) Interleukin-18 stimulates fibronectin expression in primary human cardiac fibroblasts via PI3K-Akt-dependent NF-kappaB activation. J Cell Physiol 215: 697-707. doi:10.1002/jcp.21348. PubMed: 18064631. 18064631

[21] Kubota T, Bounoutas GS, Miyagishima M, Kadokami T, Sanders VJ et al. (2000) Soluble tumor necrosis factor receptor abrogates myocardial inflammation but not hypertrophy in cytokine-induced cardiomyopathy. Circulation 101: 2518-2525. doi:10.1161/01.CIR.101.21.2518. PubMed: 10831527. 10831527

[22] Reifenberg K, Lehr HA, Torzewski M, Steige G, Wiese E et al. (2007) Interferon-gamma induces chronic active myocarditis and cardiomyopathy in transgenic mice. Am J Pathol 171: 463-472. doi:10.2353/ajpath.2007.060906. PubMed: 17556594. 17556594 PMC1934522

[23] Bilate AM, Salemi VM, Ramires FJ, de Brito T, Silva AM et al. (2003) The Syrian hamster as a model for the dilated cardiomyopathy of Chagas' disease: a quantitative echocardiographical and histopathological analysis. Microbes Infect 5: 1116-1124. doi:10.1016/j.micinf.2003.07.001. PubMed: 14554253. 14554253

[24] Zicker F, Smith PG, Netto JC, Oliveira RM, Zicker EM (1990) Physical activity, opportunity for reinfection, and sibling history of heart disease as risk factors for Chagas' cardiopathy. Am J Trop Med Hyg 43: 498-505. PubMed: 2240374. 2240374 10.4269/ajtmh.1990.43.498

[25] Ramasawmy R, Cunha-Neto E, Fae KC, Martello FG, Müller NG et al. (2006) The monocyte chemoattractant protein-1 gene polymorphism is associated with cardiomyopathy in human chagas disease. Clin Infect Dis 43: 305-311. doi:10.1086/505395. PubMed: 16804844. 16804844

[26] Cunha-Neto E, Nogueira LG, Teixeira PC, Ramasawmy R, Drigo SA et al. (2009) Immunological and non-immunological effects of cytokines and chemokines in the pathogenesis of chronic Chagas disease cardiomyopathy. Mem Inst Oswaldo Cruz 104 Suppl 1: 252-258. doi:10.1590/S0074-02762009000900032. PubMed: 19753481. 19753481

[27] Teixeira PC, Frade AF, Nogueira LG, Kalil J, Chevillard C et al. (2012) Pathogenesis of Chagas disease cardiomyopathy. World Journal-- Clinical Infectious Diseases 2: 39-53. doi:10.5495/wjcid.v2.i3.39.

[28] Ahmad F, Seidman JG, Seidman CE (2005) The genetic basis for cardiac remodeling. Annu Rev Genomics Hum Genet 6: 185-216. doi:10.1146/annurev.genom.6.080604.162132. PubMed: 16124859. 16124859

[29] Dellefave L, McNally EM (2010) The genetics of dilated cardiomyopathy. Curr Opin Cardiol 25: 198-204. doi:10.1097/HCO.0b013e328337ba52. PubMed: 20186049. 20186049 PMC2939233

[30] Lu MH, DiLullo C, Schultheiss T, Holtzer S, Murray JM et al. (1992) The vinculin/sarcomeric-alpha-actinin/alpha-actin nexus in cultured cardiac myocytes. J Cell Biol 117: 1007-1022. doi:10.1083/jcb.117.5.1007. PubMed: 1577864. 1577864 PMC2289484

[31] Gregorio CC (1997) Models of thin filament assembly in cardiac and skeletal muscle. Cell Struct Funct 22: 191-195. doi:10.1247/csf.22.191. PubMed: 9113406. 9113406

[32] Olson TM, Michels VV, Thibodeau SN, Tai YS, Keating MT (1998) Actin mutations in dilated cardiomyopathy, a heritable form of heart failure. Science 280: 750-752. doi:10.1126/science.280.5364.750. PubMed: 9563954. 9563954

[33] Jiang HK, Qiu GR, Li-Ling J, Xin N, Sun KL (2010) Reduced ACTC1 expression might play a role in the onset of congenital heart disease by inducing cardiomyocyte apoptosis. Circ J 74: 2410-2418. doi:10.1253/circj.CJ-10-0234. PubMed: 20962418. 20962418

[34] Tostes S Jr, Rocha-Rodrigues D, de Araujo Pereira G, Rodrigues V (2005) Myocardiocyte apoptosis in heart failure in chronic Chagas' disease. Int J Cardiol 99: 233-7. 15749181 10.1016/j.ijcard.2004.01.026

[35] Foo RS, Mani K, Kitsis RN (2005) Death begets failure in the heart. J Clin Invest 115: 565-571. doi:10.1172/JCI24569. PubMed: 15765138. 15765138 PMC1052022

[36] Bilate AM, Teixeira PC, Ribeiro SP, Brito Td, Silva AM et al. (2008) Distinct outcomes of Trypanosoma cruzi infection in hamsters are related to myocardial parasitism, cytokine/chemokine gene expression, and protein expression profile. J Infect Dis 198: 614-623. doi:10.1086/590347. PubMed: 18598198. 18598198

[37] Jorge MT, Macedo TA, Janones RS, Carizzi DP, Heredia RA et al. (2003) Types of arrhythmia among cases of American trypanosomiasis, compared with those in other cardiology patients. Ann Trop Med Parasitol 97: 139-148. doi:10.1179/000349803235001561. PubMed: 12803869. 12803869

[38] van Veldhuisen DJ, Linssen GC, Jaarsma T, van Gilst WH, Hoes AW et al. (2013) B-type natriuretic peptide and prognosis in heart failure patients with preserved and reduced ejection fraction. J Am Coll Cardiol 61: 1498-1506. doi:10.1016/j.jacc.2012.12.044. PubMed: 23500300. 23500300

[39] Januzzi JL Jr (2013) Natriuretic peptides, ejection fraction, and prognosis: parsing the phenotypes of heart failure. J Am Coll Cardiol 61: 1507-1509. doi:10.1016/S0735-1097(13)61507-7. PubMed: 23500284. 23500284

[40] Teixeira PC, Santos RH, Fiorelli AI, Bilate AM, Benvenuti LA et al. (2011) Selective decrease of components of the creatine kinase system and ATP synthase complex in chronic Chagas disease cardiomyopathy. PLoS Negl Trop. Drosophila Inf Service 5: e1205. 10.1371/journal.pntd.0001205 PMC3125151 21738806

[41] Schmittgen TD, Livak KJ (2008) Analyzing real-time PCR data by the comparative C(T). Methods - Nat Protoc 3: 1101-1108. doi:10.1038/nprot.2008.73. 18546601

[42] Barretto AC, Arteaga E, Mady C, Ianni BM, Bellotti G et al. (1993) Male sex. Prognostic factor in Chagas' disease. Arq Bras Cardiol 60: 225-227. PubMed: 8311729. 8311729

[43] Faé KC, Drigo SA, Cunha-Neto E, Ianni B, Mady C et al. (2000) HLA and beta-myosin heavy chain do not influence susceptibility to Chagas disease cardiomyopathy. Microbes Infect 2: 745-751. doi:10.1016/S1286-4579(00)00501-3. PubMed: 10955954. 10955954

[44] Basquiera AL, Sembaj A, Aguerri AM, Omelianiuk M, Guzmán S et al. (2003) Risk progression to chronic Chagas cardiomyopathy: influence of male sex and of parasitaemia detected by polymerase chain reaction. Heart 89: 1186-1190. doi:10.1136/heart.89.10.1186. PubMed: 12975414. 12975414 PMC1767891

[45] Nyholt DR (2004) A simple correction for multiple testing for single-nucleotide polymorphisms in linkage disequilibrium with each other. Am J Hum Genet 74: 765-769. doi:10.1086/383251. PubMed: 14997420. 14997420 PMC1181954

[46] Nyholt DR (2005) Evaluation of Nyholt's procedure for multiple testing correction - author's reply. Hum Hered 60: 61-62. doi:10.1159/000087919. PubMed: 16137994. 16137994

[47] Isnard A, Kouriba B, Doumbo O, Chevillard C (2011) Association of rs7719175, located in the IL13 gene promoter, with Schistosoma haematobium infection levels and identification of a susceptibility haplotype. Genes Immun 12: 31-39. PubMed: 20861864. 20861864 10.1038/gene.2010.43

[48] Beall CJ, Sepanski MA, Fyrberg EA (1989) Genetic dissection of Drosophila myofibril formation: effects of actin and myosin heavy chain null alleles. Genes Dev 3: 131-140. doi:10.1101/gad.3.2.131. PubMed: 2714648. 2714648

[49] Krüger M, Linke WA (2009) Titin-based mechanical signalling in normal and failing myocardium. J Mol Cell Cardiol 46: 490-498. doi:10.1016/j.yjmcc.2009.01.004. PubMed: 19639676. 19639676

[50] Knoll R, Buyandelger B, Lab M (2011) The sarcomeric Z-disc and Z-discopathies. J Biomed Biotechnol 2011: 569628. 22028589 10.1155/2011/569628 PMC3199094

[51] Pulkki KJ (1997) Cytokines and cardiomyocyte death. Ann Med 29: 339-343. doi:10.3109/07853899708999358. PubMed: 9375993. 9375993

[52] Mocelin AO, Issa VS, Bacal F, Guimarães GV, Cunha E et al. (2005) The influence of aetiology on inflammatory and neurohumoral activation in patients with severe heart failure: a prospective study comparing Chagas' heart disease and idiopathic dilated cardiomyopathy. Eur J Heart Fail 7: 869-873. doi:10.1016/j.ejheart.2004.10.014. PubMed: 16043406. 16043406

