# Supplementary figures and images for: Polymorphism in the Alpha Cardiac Muscle Actin 1 Gene Is Associated to Susceptibility to Chronic Inflammatory Cardiomyopathy
Source: PLoS One. 2013 Dec 19;8(12):e83446. doi: 10.1371/journal.pone.0083446 (PMC3868584; doi:10.1371/journal.pone.0083446)

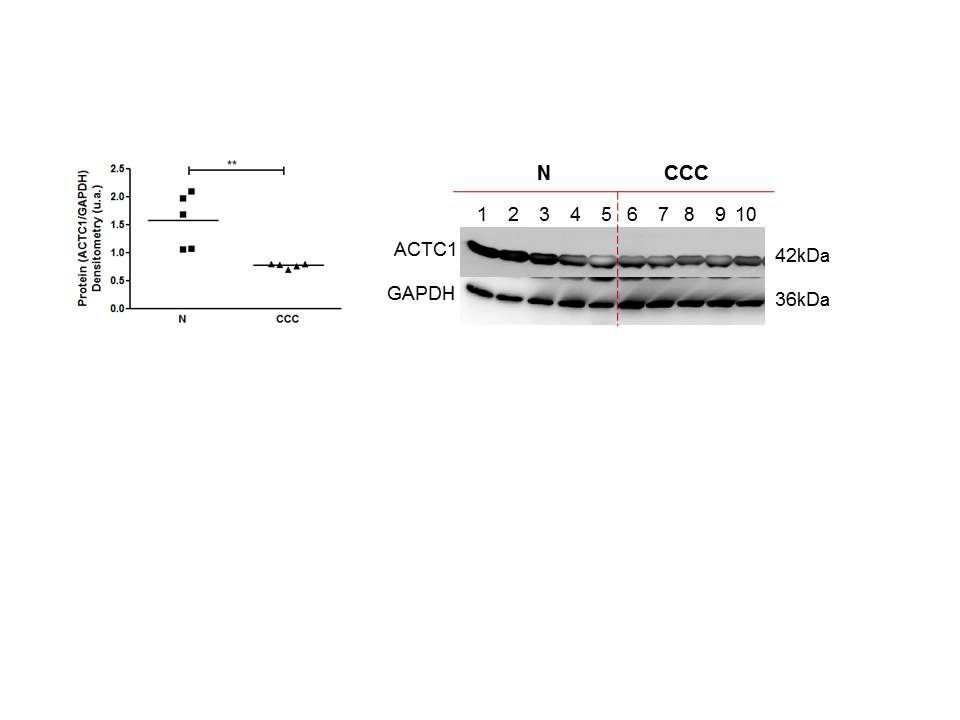

Supplement: Figure S1 — Relative quantification of alpha-cardiac actin 1 (ACTC1) by immunoblotting (duplicate analysis). Myocardial samples were obtained from the left ventricular free wall of the hearts of patients with severe CCC and end-stage heart failure, at the time of heart transplantation. Samples from five hearts from CCC patients (at least two positive results in three independent anti-T. cruzi serology tests, as indicated above), and from healthy hearts from organ donors not used for transplantation for technical reasons were used. Immunoblotting and protein quantification were done in duplicate. The immunoblot and the protein quantification result of the second experiment are presented here. The central line represents the median. Representative results from two experiments are shown here. A Mann-Whitney test was performed and differences were considered significant if P<0.001. (TIF) [file pone.0083446.s001.tif]
