# Supplementary material for: Polymorphism in the Alpha Cardiac Muscle Actin 1 Gene Is Associated to Susceptibility to Chronic Inflammatory Cardiomyopathy
Source: PLoS One. 2013 Dec 19;8(12):e83446. doi: 10.1371/journal.pone.0083446 (PMC3868584; doi:10.1371/journal.pone.0083446)
Supplement: Table S1 — Characteristics of heart sample donors. EF: Ejection Fraction (reference value: ≥55%), LVDD: Left Ventricular Diastolic Diameter (reference value: 39-53mm). N: individuals without cardiomyopathies, CCC: chronic Chagas disease cardiomyopathy. nd: not done. (DOCX) [file pone.0083446.s002.docx]

**Table S1** Characteristics of heart sample donors.

| ***Sample ID*** | ***Etiology*** | ***Gender*** | ***Age*** | ***EF*** | ***LVDD*** |
| --- | --- | --- | --- | --- | --- |
| 1 | N | M | 17 | nd | nd |
| 2 | N | M | 46 | nd | nd |
| 3 | N | M | 40 | nd | nd |
| 4 | N | M | 22 | nd | nd |
| 5 | N | M | 28 | nd | nd |
| 6 | CCC | M | 50 | 11% | 82 |
| 7 | CCC | M | 58 | 29% | 64 |
| 8 | CCC | M | 57 | 29% | 71 |
| 9 | CCC | M | 59 | 17% | 64 |
| 10 | CCC | M | 28 | 21% | 68 |

EF: Ejection Fraction (reference value: ≥55%), LVDD: Left Ventricular Diastolic Diameter (reference value: 39-53mm). N: individuals without cardiomyopathies, CCC: chronic Chagas disease cardiomyopathy. nd: not done.
